# Quality Preservation and Shelf-Life Extension of Prickly Pear (*Opuntia ficus-indica* L. *Mill*) Using Edible Coatings

**DOI:** 10.3390/foods14020161

**Published:** 2025-01-08

**Authors:** Carolina Rodrigues, Cariny Polesca, Isabela Bicalho, Victor Gomes Lauriano Souza, Isabel Coelhoso, Ana Luísa Fernando

**Affiliations:** 1 MEtRICs, Departamento de Química, NOVA School of Science and Technology (NOVA FCT), Universidade NOVA de Lisboa, Campus de Caparica, 2829-516 Caparica, Portugal; cpe.rodrigues@campus.fct.unl.pt (C.R.); polescacariny@gmail.com (C.P.); isabela.enq@gmail.com (I.B.); v.souza@fct.unl.pt (V.G.L.S.); 2 CICECO—Aveiro Institute of Materials, Department of Chemistry, University of Aveiro, 3810-193 Aveiro, Portugal; 3 School of Chemical Engineering, University of Campinas, Av. Albert Einstein 500, Campinas 13083-852, SP, Brazil; 4 LAQV-REQUIMTE, Departamento de Química, NOVA School of Science and Technology (NOVA FCT), Universidade NOVA de Lisboa, Campus de Caparica, 2829-516 Caparica, Portugal; imrc@fct.unl.pt

**Keywords:** prickly pears, edible coatings, quality preservation, shelf life

## Abstract

Prickly pear consumption is increasing across the world due to its rich variety of nutrients and bioactive compounds. Yet, it is a seasonal and highly perishable fruit, and the application of edible coatings emerges as an alternative to extend its shelf life. In this work, the effects of alginate, starch, chitosan, and pectin as coatings on the physicochemical, bioactive, microbiological, and textural properties of two prickly pear varieties (orange and red), kept under refrigeration (5 ± 2 °C) were evaluated for 6 weeks. Coatings proved to be helpful in the maintenance of the fruits’ color and textural properties, especially when pectin was applied. Overall, starch and chitosan can be considered the most effective coatings in preserving the quality of prickly pears among the options studied. A lower weight loss (8–10%) in fruits was achieved when starch and chitosan were applied, while in control fruits (without coating), the loss was 18–23%. Starch and chitosan also contributed to preserving the bioactivity of red fruits and showed good results in the preservation of total phenolic content in the orange fruits. In addition, starch and chitosan coatings also presented the best performance for the reduction of microbial contamination (both yeasts and molds and total mesophilic aerobic microorganisms). These findings highlight the role of edible coatings in preserving prickly pears, for a longer period, meeting consumers’ demand for fresh fruit.

## 1. Introduction

*Opuntia ficus-indica* L. Mill (OFI) is a native plant from Mexico belonging to the *Cactaceae* family and is currently well-established in several countries of the Mediterranean region such as Morocco, Tunisia, Italy, Spain, and Portugal [1,2], as well as in Mexico and other countries of Latin America and Africa [3,4]. OFI is known for its large pads (cladodes) but mostly for its fruits, denominated as cactus pears or prickly pears. The fruits are succulent, colorful, spiny, and rich in a variety of nutrients such as sugars, minerals, fibers, and bioactive compounds (e.g., ascorbic acid, betalains, flavonoids, phenols) [5,6]. The recent growth in demand for this fruit is associated with an increase in consumers’ healthier lifestyle choices [7]. The prickly pears are generally consumed as fresh fruits (in nature) or as minimally processed products [8].

Despite such positive characteristics, the prickly pear is seasonal (harvested during the summer) and very perishable due to its high metabolic activity and possible physical damage that can occur during harvesting and transportation [9,10]. Thus, the window time from harvesting to consumption is noticeably short, and post-harvesting preserving methods should be applied to extend its shelf life to enable its availability for prolonged periods [11].

The seasonality of prickly pears raises the necessity of studying the maximum storage duration of whole fruits to ensure that their nutritional and sanitary qualities remain uncompromised before consumption or minimal processing [12]. The prickly pear is classified as a non-climacteric fruit, and the most common post-harvesting technique used for preservation is refrigerated storage with temperatures between 5 to 8 °C for 2–5 weeks [12,13,14]. During refrigeration storage, the fruits are very susceptible to suffering chilling injury when temperatures lower than 5 °C are reached, diminishing their shelf life and causing surface pitting and browning [13,15,16]. As an alternative or as a refrigeration complement, other postharvest treatments such as chemical treatments, intermittent warming, controlled atmosphere, film wrapping, or edible coatings have been used to maintain cactus pears’ quality and prolong their shelf life [10,17,18].

Edible coating is, by definition, a thin layer of edible material applied to the surface of foods in addition to or as a substitution for commonly used coating materials [19]. It must also be acceptable to consumers as retaining the product’s original taste, texture, and appearance [19,20]. Edible coatings are obtained by the application of the coating suspension on the surface of the fruit by dipping the fruit into the suspension or by spraying the coating on the fruit’s surface [21]. The coating acts as a semi-permeable barrier that aims to protect the fruits against water loss, spoilage, and color and texture changes and to reduce the respiration rate [22,23]. Edible coatings are generally made from eco-friendly sources that are food-grade and help in the preservation of food characteristics and safety, due to their intrinsic properties or by the addition of other components into the matrix such as bioactive compounds [24,25,26]. To improve the potential of coating effectiveness, some components can be added to the coating solution such as plasticizers and cross-linking agents as well as UV-blocking agents [27,28]. Coating adhesion capacity is a crucial characteristic influenced by the surface properties of the food, the specific objectives of the coating, and the application technique used [20]. The most common technique for coating application is dipping. The dipping method depends directly on various elements, namely coating solution characteristics (e.g., density, viscosity, and surface tension), the time for immersion, the dipping velocity, and the drying conditions [29,30]. The dipping method can have some disadvantages, including irregular thick coatings that may affect fruit respiration, potential contamination of the coating solution, surface food damage, and high consumption of coating solution per food mass, which challenges scalability [20]. The effectiveness of a coating can be evaluated by monitoring the physicochemical, biochemical, and microbiological properties of the coated food throughout the storage period [25]. The most commonly used edible coatings are polysaccharides such as alginate, starch, chitosan, and pectin [27]. Polysaccharides’ coatings are characterized for having good barrier properties to gases (carbon dioxide and oxygen) but they possess high water vapor permeability and low water solubility leading to a higher swelling which directly affects the moisture loss of the fruits [20,27,30,31]. Each of them possesses particular characteristics and applications. Alginate is a linear polysaccharide extracted from brown seaweeds, and it is used as a coating due to its thickening properties, gel-forming capacity, emulsion stabilizing, and high availability [32,33]. Alginate edible coatings have been demonstrated to be effective in the preservation of strawberries [34], fresh-cut Fuji apples [35], rose apples [36], mangos [37], blueberries [32], and sweet cherries [38]. Starch is an abundant polysaccharide that can be extracted from potatoes, corn, and beans and is composed essentially of amylose and amylopectin [39]. Starch-based edible coatings are used due to their good mechanical properties, high availability, and low cost [40]. Their use on plums [41], bananas [42], fresh-cut mangoes [43], Red Crimson grapes [44], and mandarin oranges [45] was proven to contribute to extending the fruits’ shelf life. Chitosan (poly-β-(1,4)-2-amino-2-deoxy-D-glucose) is the second most abundant polysaccharide in nature, obtained from the N-deacetylation of chitin, present in the crustacean shells [46,47]. It is worth mentioning chitosan’s intrinsic antimicrobial activity, which together with its good gel and film-forming capacity, highlights this polysaccharide as a good matrix to preserve food products [47,48,49]. Its use as an edible coating contributed to the conservation of cherry tomatoes [48], peaches [49], sweet cherries [50], apples [51], and strawberries [52]. Pectin is a complex polysaccharide and is usually extracted from citrus peels and is widely used to enhance viscosity and gel-forming capacity [53]. Studies of the use of pectin edible coatings in fresh-cut peaches [54], limes [55], fresh-cut persimmons [56], strawberries [57], and plums [41,58] demonstrate that is a favorable method not only to extend fruits’ shelf life but also to maintain their nutritional value.

Chitosan, as a cationic polymer, forms stable films or coatings through its inherent intermolecular interactions, such as hydrogen bonding and electrostatic forces. Starch, on the other hand, creates films via hydrogen bonding and gelatinization processes during its structural transition in the presence of water and heat. In contrast, anionic polymers like sodium alginate and pectin require cross-linking agents to form stable film networks. These agents, typically divalent cations such as Ca^2+^, help create cohesive and durable coatings. Calcium chloride is widely used as a cross-linking agent in food applications due to its recognized safety and effectiveness in enhancing the structural and functional properties of edible films [59].

Some previous studies applied coatings to prickly pear preservation, focusing on the use of natural compounds to extend the fruits’ shelf life. As an example, the use of 1% chitosan in a 1% (*v*/*v*) solution of acetic acid was demonstrated to be effective in delaying the weight loss and integrity of the fruits. Moreover, a 1% chitosan coating in 1% (*v*/*v*) and 2.5% (*v*/*v*) acetic acid solutions led to the maintenance of bioactive compounds present in prickly pears [17]. Additionally, a concentration of 3% cassava starch as a coating solution for prickly pears was efficient in maintaining fruit quality for 25 days [60].

In this context, the objective of this study was to evaluate the effect of four edible coatings (alginate, starch, chitosan, and pectin) on the physicochemical, bioactive, textural, and microbiological properties of two cactus pear varieties (orange and red) for 6 weeks. The weight loss, moisture, pH, titratable acidity, total soluble solids, color, and textural properties of the fruits, coated and uncoated, and kept under refrigeration (5 ± 2 °C) for 6 weeks were monitored. The effect of the coatings on the bioactive properties (total phenolic compounds, antioxidant activity, ascorbic acid, and betalain content) was also evaluated. Finally, the total mesophilic aerobic microorganisms, yeasts, and mold growth were measured to evaluate the effects of the different coatings on the microbiological quality of prickly pear peel and pulp. Since it is a seasonal fruit, the main motivation for this study is to understand the extension of cactus pear shelf life by using these edible coatings in order to meet the industry’s and consumers’ demands. Even though there are several studies pointing to the use of polysaccharide coatings to extend prickly pear shelf life, an extensive evaluation and comparison among various coatings has not yet been carried out. Once the exact efficacy of the coatings depends on the unique surface properties, respiration rates, and physicochemical characteristics of the tested fruits, this work serves as a valuable reference on the effectiveness of the different formulations.

## 2. Materials and Methods

### 2.1. Materials and Reagents

#### 2.1.1. Biomass Material

OFI prickly pears (orange and red varieties) were harvested in the period of September 2019 and kindly provided by the *Pepe Aromas* company, located in Vale Pereiro, Évora, Portugal. The fruits were harvested and brushed and the spikes were removed in the company facilities, and then they were brought in cardboard boxes under refrigeration to the NOVA School of Science and Technology facilities. In the laboratory, prickly pears were washed with water and left to dry for further use. From a total of 400 fruits (200 from each variety) the sample selection was based on the size and appearance and 62 prickly pears from each variety were selected to proceed with the experiment.

#### 2.1.2. Reagents

Chitosan with high molecular weight (31–37 kDa) and with a deacetylation degree of 75% and pectin from citrus peel (galacturonic acid of ≥74% of dried basis) were used as a polymeric matrix and purchased from Sigma-Aldrich (St. Louis, MO, USA). Corn starch and sodium alginate were also used as polymeric matrices supplied from Sosa (Barcelona, Spain). All chemical reagents were of analytical reagent grade and were used as purchased. Glacial acetic acid (≥99.8% purity) and gallic acid (99% purity) were purchased from Alfa Aesar (Karlsruhe, Germany), while Folin-Ciocalteau reagent, sodium carbonate anhydrous (99.5% purity), sodium hydroxide (≥99% purity), and sodium chloride (99.5% purity) were obtained from Panreac (Barcelona, Spain). Calcium chloride (≥97% purity) was purchased from Honeywell (Charlotte, NC, USA). Ethanol absolute (≥99.8% purity), 2,2-diphenyl-1-picrylhydrazyl (DPPH) was supplied by Sigma-Aldrich (USA). The 2,6-dichlorophenol-indophenol was purchased from Merck (Darmstadt, Germany). All microbiological reagents were purchased from Biokar (Allonne, France): Dichloran Rose Bengale Chloramphenicol (DRBC), tryptone, and plate count agar (PCA). All the water used was purified using a Milli-Q system (Millipore, St. Louis, MO, USA).

### 2.2. Coatings

#### 2.2.1. Preparation of Coatings Solution

Coating solutions were prepared by dissolving alginate (2% *w*/*v*), pectin (2% *w*/*v*), and starch (2% *w*/*v*) in distilled water under controlled stirring and heating (70 °C for alginate and pectin and 100 °C for starch) until the mixtures were homogenized. Then, the solutions were cooled to room temperature. The chitosan coating solution was prepared by dissolving 2% (*w*/*v*) in 1% (*v*/*v*) of glacial acetic acid solution under continuous stirring overnight at room temperature. The concentration used for the coating solution was standardized and applied based on previous experiments and literature [35,61,62,63,64].

#### 2.2.2. Fruit Coatings

Previously washed fruits were submerged for 2 min in the coating solution. Afterward, the fruits were placed on a grid to drain and dry. In the case of fruits coated with sodium alginate and pectin solutions, fruits were drained and then dipped (2 min) in a calcium chloride (2% *w*/*v*) solution for cross-linking of the coating material. The fruits were then placed on a grid to drain and dry. Fruits were stored in petri dishes, under refrigeration (5 ± 2 °C) for 6 weeks. As indicated previously, anionic polymers such as sodium alginate and pectin require cross-linking agents to form stable film networks. For this reason, calcium chloride was used as a cross-linking agent.

### 2.3. Prickly Pear Evaluation

The prickly pears’ peel and pulp were characterized every week for 6 weeks. In this study, we intentionally extended the storage duration to 6 weeks, to assess the effect of edible coatings on prickly pears beyond the typical maximum storage time (2–5 weeks) to provide insights into their potential for extending the shelf life of the fruits. The following methods were applied.

#### 2.3.1. Physical and Chemical

##### Moisture Content and Weight Loss

Moisture content was evaluated according to the AOAC (Association of Official Analytical Chemists) method [65]. Weight loss was determined gravimetrically by the difference of the initial weight (0 days), and the fruit weight at weeks 1, 2, 3, 4, 5, and 6. The results were calculated according to Equation (1):

(1) $$Weight\;loss\left(\%\right)=\left(\frac{a-b}{a}\right)\times 100$$

where *a* is the initial weight and *b* is the final weight for each week.

##### pH, Titratable Acidity, and Total Soluble Solids

The pH was measured by using a potentiometer HI 2210 (Hanna Instruments, Woonsocket, RI, USA). Titratable acidity was determined according to the AOAC (Association of Official Analytical Chemists) method [65]. The analysis was performed by mixing 5 g of sample with deionized water and determined by titration with NaOH 0.02 N. The results were calculated according to Equation (2) and expressed as %(*w*/*w*) of g of citric acid equivalent.

(2) $$Titratable\;acidity=\left(\frac{v_{NaOH}\times {meq}_{citric\;acid}\times N_{NaOH}}{W\times 1000}\right)\times 100$$

where *v_NaOH_* is the volume of NaOH used in the titration, *meq_citric acid_* is the equivalent value for citric acid (70.7 g), *N_NaOH_* is the normality of NaOH, and the *W* is the weight (g) of the sample used.

Total soluble solids were determined by refractometry using an analogic and manual refractometer HI 96801 (Hanna Instruments, USA) and expressed in °Brix.

#### 2.3.2. Color and Textural Properties

##### Color

Color was measured by the determination of the CIE (International Commission on Illumination)—L*a*b* coordinates using a CR 410 colorimeter (Minolta Co., Tokyo, Japan). L* represents lightness, where L = 0 (black) to L = 100 (white), a* represents redness from −(green) to +(red), and b* from −(blue) to +(yellow). The L*a*b* coordinates allowed calculation of the hue angle (h°) according to the following equation (Equation (3)) [66]:

(3) $$h{}^{\circ}=arc{tan}^{-1}\left(\frac{b^{*}}{a^{*}}\right),\;ifa^{*}>0;\;arc{tan}^{-1}\left(\frac{b^{*}}{a^{*}}\right)+180,\;ifa^{*}<0$$

##### Textural Properties

The mechanical properties of prickly pear were evaluated by uniaxial compression test using a texturometer CT3 4500 (Ametel Brookfield, Middleborough, MA, USA). Stress at failure values of the samples were determined using a 50.8 mm diameter cylindrical with a rounded edge 20 mm long probe at a compression speed of 1 mm/s until it penetrates 6 mm in the cubic sample. The fruits were peeled and two samples of 20 × 20 × 20 mm of each fruit were collected from the equatorial region (center) of the prickly pear interior [17].

#### 2.3.3. Bioactive Properties

To access the bioactive properties, 10 g of prickly pear pulp was mixed with 100 mL of deionized water for 30 min under constant stirring. After that time, the samples were filtered and used in the analysis described below.

##### Ascorbic Acid Content

The ascorbic acid content was determined by the 2,6-dichlorophenol indophenol titrimetric method according to AOAC method [65].

##### Total Phenolic Content (TPC)

Total phenolic content was determined according to the Folin–Ciocalteau method [67] with slight modifications. Briefly, 1 mL of diluted prickly pear pulp was mixed with 3 mL of Milli-Q water and 0.25 mL of Folin–Ciocalteau reagent. After 5 min of incubation at room temperature, 0.75 mL of sodium carbonate solution 20% (*w*/*v*) was added, stirred, and stored in the dark for 60 min. The absorbance of the mixture was measured in a UV-Vis spectrophotometer E-1000UV (Peak Instruments, Westford, MA, USA) at 760 nm. Gallic acid was used to elaborate a calibration curve with solutions from 0–120 mg/L. TPC was expressed in mg of gallic acid equivalent (GAE)/100 g.

##### Antioxidant Activity

Antioxidant activity was measured by the DPPH method (2,2-diphenyl-1-picrylhydrazyl) free radical scavenging assay [68]. Briefly, 1 mL of the diluted prickly pear pulp was mixed with 3 mL of 60 μmol/L DPPH ethanolic solution, then the mixture was kept in the dark for 20 min. The absorbance was measured at 517 nm in a UV-Vis spectrophotometer E-1000UV (Peak Instruments, USA)**.** The results were calculated as the Trolox equivalent antioxidant capacity (TEAC), using a calibration curve and expressed in mg of Trolox equivalent/mL.

##### Betalain Content

For betalain content (BC) determination, aqueous extracts from pulp were performed. Betaxanthins (yellow pigments) and betacyanins (red pigment) content were calculated as described in Equation (4) [69,70]:

(4) $$BC\;content\left(mg/L\right)=\left(\frac{Abs\times DF\times MW\times 1000}{\varepsilon \times l}\right)$$

where *Abs* is the absorbance measured, *DF* is the dilution factor (1:100), *MW* is the molecular weight (g/mol), *l* is the path length (1 cm), and *ε* the molar extinction coefficient (L/(mol/cm).

For betaxanthin and betacyanin quantification, the betanin and indicaxanthin molecular weights and molar extinction coefficients were: betanin (*MW* = 308 g/mol; *ε* = 48,000 L/(mol.cm)) and indicaxanthin (*MW* = 550 g/mol; *ε* = 60,000 L/(mol.cm)). The absorbance of betaxanthins and betacyanins was measured at λ = 480 nm and λ = 535 nm, respectively, in a UV-Vis spectrophotometer E-1000UV (Peak Instruments, USA).

#### 2.3.4. Microbiological Growth

Total mesophilic aerobic microorganism (TMAM) and yeast and mold growth were measured to evaluate the microbiological quality of prickly pear peel and pulp, according to ISO 4833-1:2013 [71] and ISO 21527-1:2008 [72], respectively. TMAM counts were performed in PCA after incubation at 30 °C for 72 h. Mold and yeast counts were performed in DRBC after incubation at 25 °C for 128 h. The results were expressed as log CFU (colony forming units)/g of sample.

### 2.4. Statistical Analysis

All experiments were conducted using a completely randomized design with three replications. Statistical analysis of the data was performed through a one-way analysis of variance (ANOVA) using Software OriginLab (version 8.5) (Northampton, MA, USA), and when the ANOVA was significant (*p* < 0.05), differences among mean values were processed by the Tukey test. Significance was defined at *p* < 0.05.

## 3. Results and Discussion

**Figure 1:**
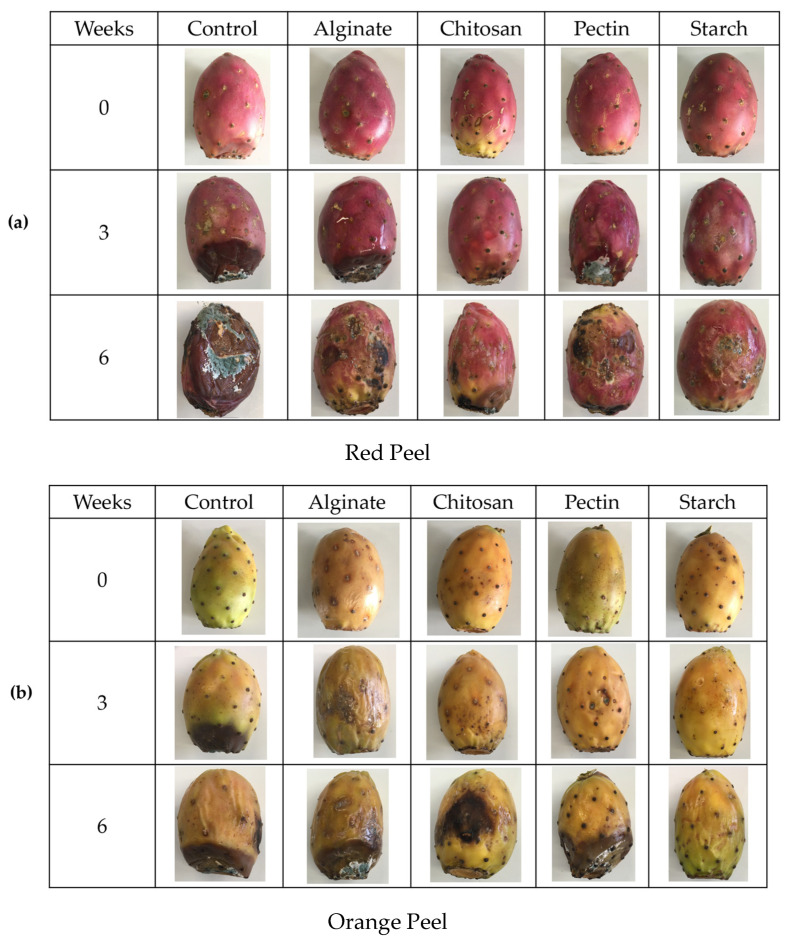
Visual aspect of red (**a**) and orange (**b**) prickly pears at initial time, 3, and 6 weeks of the experiment.

The visual aspect of both red and orange prickly pear fruits, with and without coatings, during the six-week study, is presented in Figure 1.

### 3.1. Weight Loss

**Figure 2:**
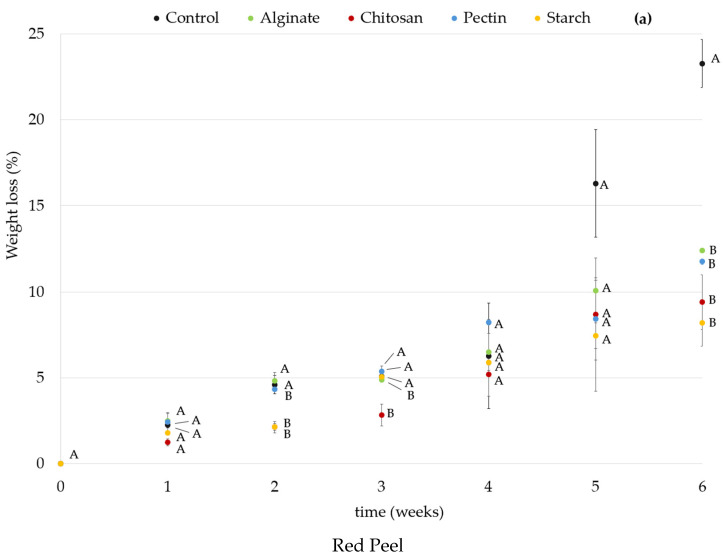

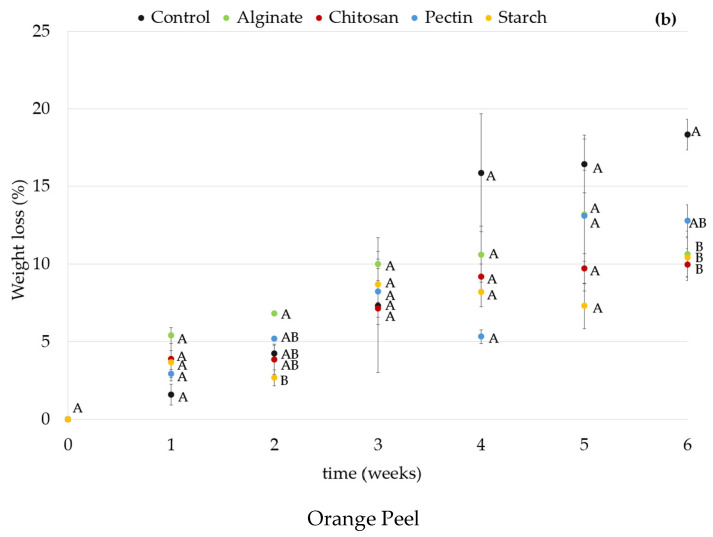
Weight loss (%) results of red (**a**) and orange (**b**) prickly pear during storage time. ^(A–B)^: Different upper case letters indicate significant differences (*p* < 0.05) between coatings at the same time of the experiment.

The weight loss percentages of red and orange prickly pears over the weeks are displayed in Figure 2. Fruit weight loss is related to water loss derived from metabolic processes of respiration and transpiration that occur in fruits during storage [37,73]. Coatings can help in reducing weight loss by lowering respiration rate, improving the fruits’ appearance, and extending their shelf life [20]. Polysaccharide edible coatings have good barrier properties to gases but high water vapor permeability, which can affect weight loss [30]. In general, an increase in weight loss during the weeks was verified for all the fruits studied. The use of coatings significantly reduced the weight loss of prickly pears when compared with controls (*p* < 0.05), at the end of the 6 weeks. However, no significant differences were observed between control and pectin-coated fruits in the orange variety at that time. The most successful coatings in the reduction of weight loss were starch (8.2%) in red fruits and chitosan (9.9%) in orange fruits. These results were in agreement with Batista Silva et al., (2018) who reported that a decrease in weight loss was also observed in chitosan-coated guava when compared with the control [74]. Starch as a coating material was shown to be effective in reducing weight loss in guava [75] and in Red Crimson grapes [44] when compared to uncoated fruits. Regarding fresh-cut prickly pears, similar behavior was observed by Liguori et al., showing an increase in weight loss for uncoated fresh-cut prickly pears compared to mucilage-coated fruits kept under similar conditions for 9 days [76]. Even though polysaccharide edible coatings show high water vapor permeability, the solvent used in the coating solution can influence that property and particularly in chitosan, the use of acetic acid, as in this study, can help in the reduction of water vapor permeability, which has a positive influence on weight loss [39].

### 3.2. Moisture Content, pH, Titratable Acidity, and Total Soluble Solids

The results obtained on the moisture content, pH, titratable acidity, and total soluble solids are presented in Table 1 and Table 2 for the red and orange varieties, respectively.

Fruits usually have a high moisture content, making them highly perishable and susceptible to microbial contamination [77]. Thus, the use of edible coatings can be helpful in maintaining their shelf life by playing a role as a barrier against moisture losses, gas exchanges, and mechanical damages, to mention a few [19]. The initial moisture content of both prickly pear varieties is approximately 84%, which is similar to the values reported by Martins et al. (2023) [13]. The moisture content of the red prickly pear (Table 1) did not statistically change (*p* > 0.05) over the storage period assessed; however, comparing day 6 to day 0, it is noticeable that all fruits presented a tendency of a gradual decrease in this attribute, reflecting the water loss in fruit. Despite slight variations in the moisture content, there were no statistically significant differences (*p* > 0.05) between the control and the coated samples, independently of the coating. From the beginning of the experiment until the end, fruits lost about 9–10% of their water, and this behavior was transversal to all coated fruits and controls, showing that the use of coatings was not impactful for the preservation of water content. In the orange variety (Table 2), similar behavior was observed for both coated and uncoated fruits. Despite there being no significant differences (*p* > 0.05), the control fruits exhibited the most rapid and marked moisture loss from an initial 82.2% to 59.8% after the sixth week of storage, while the coated fruit showed final moisture of approximately 77–78%, showing that coatings had a positive effect on retaining water in orange prickly pears, which corroborates with the findings in weight loss. Comparable results were reported by Andreu-Coll et al. (2021), showing that moisture loss was more predominant in uncoated prickly pears than in alginate-coated orange prickly pears, during 10 days of storage at 4 °C [14]. Overall, a loss in the moisture content was observed for both varieties, but the losses were lower in red prickly pear and coatings did not show a high positive effect on moisture retention.

Prickly pear is considered a neutral fruit in terms of pH (5.3–7.1) and the values obtained throughout the experiment were within that range for both varieties [13,76,78]. The pH values of the coated and control red fruits varied moderately throughout the storage period. On week 2, significant differences were observed (*p* < 0.05), with chitosan-coated fruits showing the lowest results (6.18) and the control the highest (6.31). In the sixth week, alginate-coated fruits showed a significant decrease (5.53) (*p* < 0.05) when compared to the remaining coated fruits and control. Amongst all the coatings, chitosan seemed to help maintain the pH stability of the red prickly pears as well as starch. These results could be due to the intrinsic properties of chitosan that reduce microbial or enzymatic activity [30]. In the orange variety, the pH values of the fruits had slight variations during the storage period with no statistically significant differences (*p* > 0.05). These observations were also noted by Panza et al. (2022) who found no differences in pH between uncoated and alginate-coated red prickly pears after 10 days of storage [79]. In general, the pH levels of both red and orange prickly pears remained stable during storage, with slight decreases over time.

Prickly pear has usually a total soluble solid content of 12–17 °Brix [78,80]. The content of soluble solids increase is related to fruit ripening and is also responsible for the sweet taste of fruits [19]. The initial °Brix values obtained, 13.2 °Brix (red prickly pear) and 13.5 °Brix (orange prickly pear) are similar to the values reported in the literature [81,82]. The total soluble solids (TSS) content, measured in °Brix, showed some variations during the storage period. By week 6, all treatments maintained TSS values within a similar range to the initial values, with no statistically significant differences (*p* > 0.05) in both varieties. However, a notable increase in TSS was observed in control and coated red fruits, especially in the alginate-coated red fruits at week 2, which could be related to the concentration of sugars as water content decreased [83]. This temporary increase may suggest that alginate-coated fruits underwent more rapid dehydration early in the storage period compared to the other treatments, although this effect stabilized by week 6. Similar behavior was observed in grapes coated with alginate during 12 days of storage [84]. In the orange fruits, coated fruits showed more stable values, especially the alginate- and chitosan-coated fruits, maintaining a value of TSS of 13 °Brix over the time of the experiment. While there were no significant differences among coatings at most storage times (*p* > 0.05), the control showed a high variability, indicating that coatings may contribute to stabilizing TSS levels. The use of edible coatings in fruits can contribute to retard the alterations in TSS by affecting the respiratory and metabolic processes by retarding them [85]. Moreover, the alterations of this parameter can also be related to distinct stages of maturity of the fruits and the senescence phenomenon that occurred during the storage period [84].

Total titratable acidity is an important parameter directly related to the acids present in food. In food, organic acids represent a great part of the acids present and have a high influence on flavor, color, and microbial activity, amongst others [86]. The total titratable acidity at the initial time for both varieties is coincident with the values reported in the literature for fresh fruits [81,87]. In the red fruits, total titratable acidity remained relatively stable throughout the time, showing some fluctuations at specific time points. Alginate-coated fruits showed statistically significant differences (*p* < 0.05), especially between week 0 and week 6 when this coating showed the highest value of TTA (0.08% of citric acid (wt/wt)), which is in agreement with the pH value at that time of the experiment. In contrast, chitosan-coated fruits maintained lower total acidity values throughout the weeks, even though significant differences were observed (*p* < 0.05) likely due to the intrinsic antimicrobial and antifungal properties of this polysaccharide which potentially helped on the inhibition of antimicrobial and antifungal activity and thus limited acid production [88,89]. At week 6, no significant differences (*p* > 0.05) were observed between coated and uncoated fruits. In the orange fruits, values increased slightly for all coated and uncoated fruits over the time of the experiment. Some fluctuations were observed right in the first week of the experiment when significant differences (*p* < 0.05) were observed, especially between uncoated fruits and starch-coated fruits. At the end of the storage period, chitosan-coated red fruits presented the highest value of TTA (0.07% citric acid (wt/wt)) and alginate-coated the lowest (0.05% citric acid (wt/wt)) amongst the coated and uncoated fruits but with no significant differences (*p* > 0.05) between them. Alginate coating was helpful in retarding the acidity loss in plums for 35 days at 2 °C [90]. The use of cassava starch as a coating in prickly pear cv. Gigante demonstrated positive effects on delaying the total titratable acidity increment when compared to uncoated fruits [60]. The increase in TTA over time was observed in both varieties, but the red variety showed more pronounced differences between coatings. The orange variety, however, showed a slight but more uniform increase in TTA across all coatings. The increase of acidity common to both varieties may be related to the low respiratory activity at the end of storage time and highly related to fruit senescence, which could indicate the accumulation of organic acids [60].

### 3.3. Color and Textural Properties

**Figure 3:**
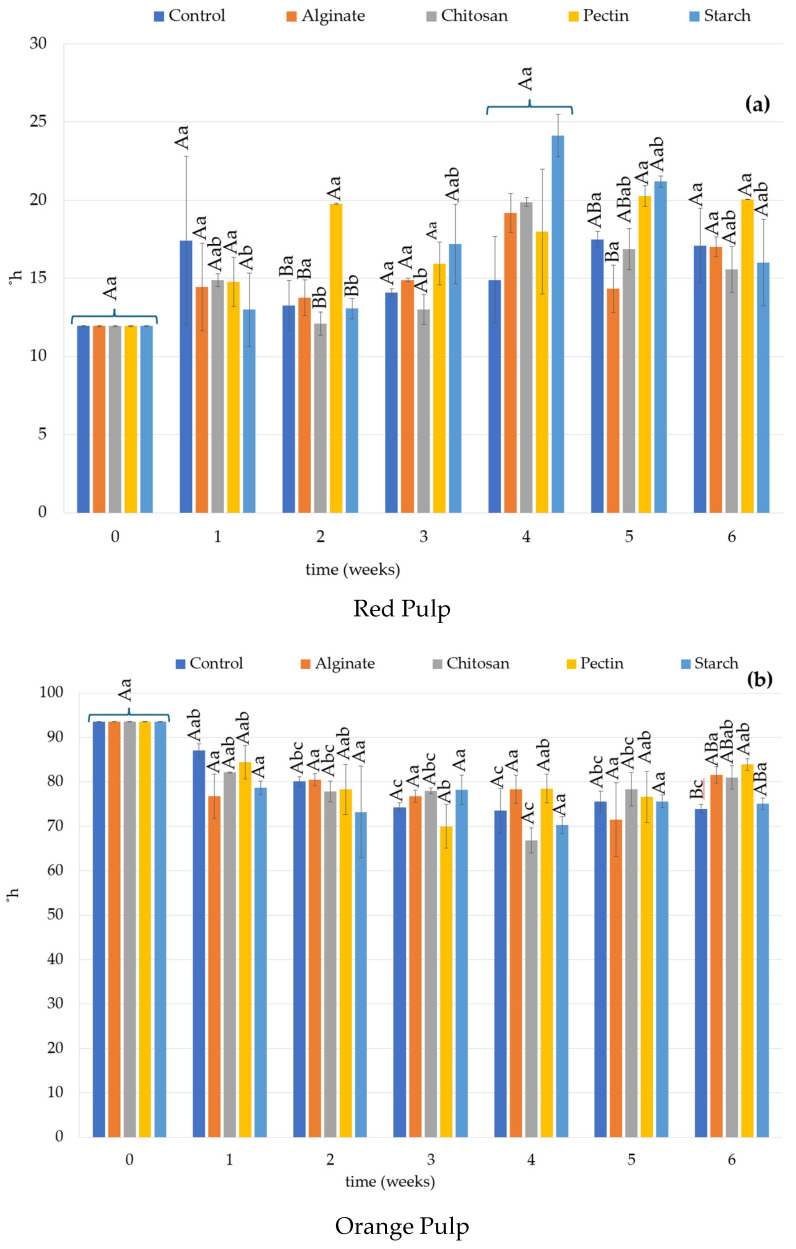
Hue angle (h°) results of red (**a**) and orange (**b**) prickly pear pulp during storage time. ^(A,B)^: Different capital letters indicate statistically significant differences (*p* < 0.05) between treatments at the same time of the experiment. ^(a–c)^: Different lower case letters indicate statistically significant differences for the same sample over time (*p* < 0.05).

**Figure 4:**
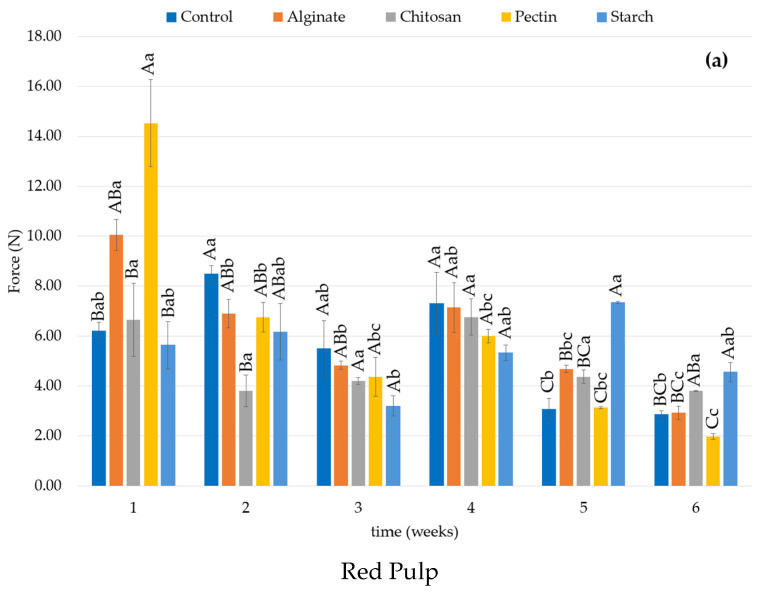

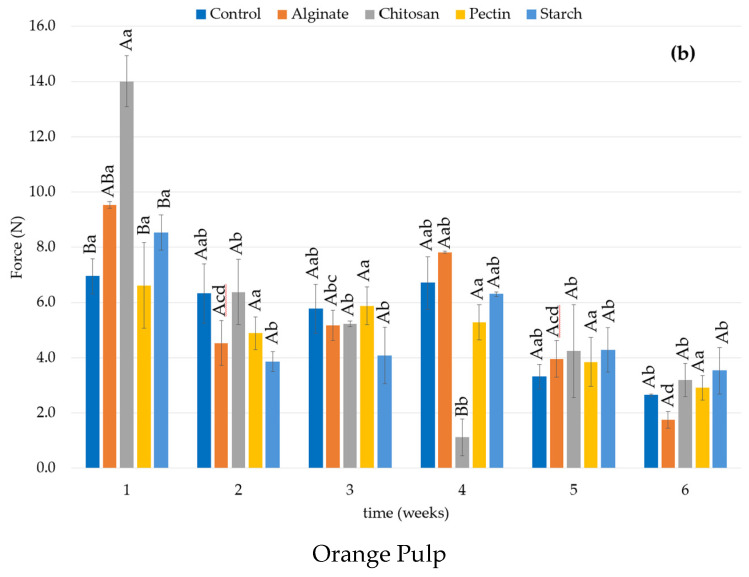
Firmness results (N) of red (**a**) and orange (**b**) prickly pear pulp during storage time. ^(A–C)^: Different capital letters indicate statistically significant differences (*p* < 0.05) between treatments at the same time of the experiment ^(a–d)^: Different lower case letters indicate statistically significant differences for the same sample over time (*p* < 0.05).

The color and textural properties of red and orange coated and uncoated prickly pears are presented in Figure 3 and Figure 4, respectively.

The color of a fruit has a major influence on its appearance and is a key factor that influences the consumers’ decision to purchase a food product [91]. Hue angle is related to ripening phenomena.

For the red prickly pears, the hue angle had some variations over time but, progressively changed from red to red-orange color (Figure 3a). At the beginning of the experiment, a value of *h*° of around 12 was obtained, slightly lower than the value of 16.26 reported by Lekhuleni et al. (2021) [81]. Chitosan- and starch-coated fruits showed significant differences over time (*p* > 0.05) while the other coated and uncoated fruits showed no difference (*p* < 0.05). Chitosan and starch contributed positively to the maintenance of the fruits’ color, while pectin led to the highest variation in hue angle value at the end of the experiment. The use of chitosan coating also had a positive effect on hue maintenance in guavas [74], and starch had the same effect in bananas [42]. The change in color from red to reddish-orange can be related to browning phenomena that possibly caused the degradation of betalains (check discussion in Section 3.4) and could also be affected by storage conditions [91].

Hue angle is related to ripening phenomena and for orange prickly pears decreases with the progression of ripening, as color changes from yellowish-orange to red-orange. The initial *h*° value obtained for the orange prickly pear was around 93.50, which is similar to the value reported by Eroglu et al. (2022) for the same variety of fruit [92]. The hue angle decreased with storage weeks irrespective of the coating (Figure 3b). The change in hue angle was significant and rapid in coated and non-coated fruits until week 2 and less rapid afterward.

In the last week of storage, orange prickly pear fruits had hue angle values in this decreasing order: pectin > alginate > chitosan > starch > control. Therefore, coatings inhibit respiration and ethylene production delaying senescence by creating a modified atmosphere around the fruit surface. The same was reported for tomatoes [93], guavas [94], mangos [95], and persimmons [96]. The decrease in hue angle was also noted in another study with *Opuntia ficus-indica* [14] and *Opuntia albicarpa* [97] under cold storage.

Firmness is a key property in the quality characteristics of fruits and vegetables [97]. Overall, the firmness of all fruits, despite the variety (orange or red) and presence of coating (Figure 4), reduced over the refrigerated storage time, however with statistical significance only for some of the treatments.

Differences in the fruits coated with different biopolymers were observed since week 1 of storage (*p* < 0.05), with superior firmness in the red fruits coated with pectin or alginate, while in the orange fruits, chitosan or alginate stood out. At the end of the refrigerated storage, at week 6, for the red prickly pears, only pectin- and alginate-coated fruits presented statistical difference from the initial firmness (*p* < 0.05), and within coatings, fruits protected with starch presented the highest firmness. In orange fruits, only pectin maintained the firmness from week 1 to week 6 (*p* > 0.05), and no differences were observed between coatings. Similar behavior was observed in prickly pears minimally processed and coated with Guar and Xanthan [98]. The authors reported a delay in the loss of fruit firmness in the initial storage days; however, after 9 days, this difference was not statistically significant, and control and coated fruits presented the same firmness. During the ripening process, a decline in firmness is expected [97]. The loss of firmness of prickly pears can be associated with the breakdown of its cell wall structure, along with enzymatic activities involving pectinmethylesterase, polygalacturonase, and cellulase, which affect pectic substances [17]. The presence of coatings may have influenced this biochemical metabolism as it retards the gas exchange (O_2_, CO_2_ and H_2_O) through the membrane formed (coating) [99].

### 3.4. Bioactive Composition

Bioactive properties are displayed in Table 3 and Table 4 for the red and orange varieties, respectively, showing the results of total phenolic compounds, antioxidant activity, ascorbic acid content, and betalains (indicaxanthin and betanin). In general, these parameters were significantly affected by storage time and coating applied.

Phenolic compounds are a group of secondary metabolites that are produced by plants and food and play an important role in protection from environmental factors such as UV light [14,28]. The initial value of total phenolic compounds for red and orange prickly pears was 772 mg GAE/100 g and 588 mg GAE/100 g, respectively. These values are within the same range as the ones reported by Valero-Galván et al. (2021) [100]. Total phenolic compound (TPC) content varied throughout storage time. There were no statistically significant differences between coatings during the entire experiment for either red or orange varieties and control fruits (*p* > 0.05). Alginate-coated red fruits showed a significant reduction (*p* < 0.05) from week 0 to week 6 (543 mg GAE/100 g). In week 5, pectin-coated orange fruits exhibited a rise in TPC (878 mg GAE/100 g) and showed significant differences (*p* < 0.05) when compared to the remaining weeks of the study. Overall, the red variety showed a higher TPC over the weeks, particularly with starch among the coatings. Thus, these results indicate that coatings helped in the mitigation of the immediate loss of phenolic compound content, their long-term efficacy varied, but no coating highly influenced the preservation of phenolic compounds. Similar behavior in coated fruits was also reported in strawberries using hydroxyethyl cellulose/sodium alginate/asparagus waste extract [101]. Moreover, cold storage conditions are reported to influence the phenolic compound content and help with the maintenance and/or increase of their content when compared to room temperature [6,14,102].

Antioxidant activity is directly related to the presence of antioxidant compounds such as phenols and ascorbic acid that can act as a protection against reactive oxygen species (ROS). Through radical scavenging, the antioxidants can help in the degradation of fruits and vegetables [28]. Overall, a decrease in antioxidant activity was observed over the weeks for both red and orange fruits. For red fruits, antioxidant activity revealed a significant decline in all treatments after the beginning of the experiment (*p* < 0.05), with the control showing the most rapid loss. Alginate-coated red fruits’ antioxidant activity was significantly reduced by week 6 (459 mg eq Trolox/mL) compared to week 0, but lower than with chitosan (560 mg eq Trolox/mL) and starch (602 mg eq Trolox/mL) and superior to the control (385 mg eq Trolox/mL), suggesting chitosan and starch are more effective in delaying the degradation of bioactive antioxidant compounds present in the fruits. The statistical differences between coatings became more evident by week 5 when starch showed the higher antioxidant activity to be statistically superior (*p* < 0.05), while the other coatings showed lower antioxidant capacity. In orange fruits, the antioxidant activity declined in all treatments after the beginning, with the control dropping drastically by week 1 (*p* < 0.05). Alginate- and starch-coated fruits showed higher antioxidant activity in the first two weeks, but by the end of the experiment, antioxidant activity was almost entirely depleted in all samples (less than 10% of the initial activity). In fact, at the end of 6 weeks, starch-coated fruits were the ones with higher antioxidant activity (460 mg eq Trolox/mL) showing no statistically significant differences when compared with the other coated fruits and the control (*p* > 0.05). Thus, starch appeared to be the best coating in preserving antioxidant activity in red and orange fruits even though rapid antioxidant degradation occurred. These results are not supported by other authors who report that edible coatings can help in the maintenance of antioxidant properties in coated fruits [84,103]; however, they corroborate with the trends observed for the contents in betalains and ascorbic acid, as discussed below.

**Table 3:** Bioactive properties results of the coated and uncoated red prickly pear pulp fruits during storage time.

Parameter	Week	Control	Alginate	Chitosan	Pectin	Starch
TPC (mg GAE/100 g)	0	772 ± 0 ^Aa^	772 ± 0 ^Aab^	772 ± 0 ^Aa^	772 ± 0 ^Aa^	772 ± 0 ^Aa^
	1	702 ± 84 ^Aa^	754 ± 65 ^Aab^	754 ± 119 ^Aa^	764 ± 5 ^Aa^	886 ± 84 ^Aa^
	2	591 ± 11 ^Aa^	640 ± 5 ^Aab^	637 ± 100 ^Aa^	605 ± 95 ^Aa^	770 ± 130 ^Aa^
	3	759 ± 86 ^Aa^	624 ± 92 ^Aab^	621 ± 138 ^Aa^	697 ± 46 ^Aa^	635 ± 38 ^Aa^
	4	924 ± 343 ^Aa^	824 ± 38 ^Aa^	683 ± 119 ^Aa^	586 ± 70 ^Aa^	764 ± 151 ^Aa^
	5	672 ± 16 ^Aa^	664 ± 35 ^Aab^	478 ± 5 ^Aa^	726 ± 76 ^Aa^	713 ± 122 ^Aa^
	6	635 ± 97 ^Aa^	543 ± 43 ^Ab^	602 ± 27 ^Aa^	554 ± 43 ^Aa^	648 ± 154 ^Aa^
Antioxidant activity (mg eq Trolox/100 mg)	0	2394 ± 526 ^Aa^	2394 ± 526 ^Aa^	2394 ± 526 ^Aa^	2394 ± 526 ^Aa^	2394 ± 526 ^Aa^
	1	1193 ± 433 ^Aa^	1543 ± 83 ^Aab^	1227 ± 67 ^Aab^	1527 ± 217 ^Aab^	1618 ± 208 ^Aa^
	2	1327 ± 67 ^Aa^	1768 ± 75 ^Aab^	1293 ± 50 ^Aab^	1410 ± 400 ^Aab^	1843 ± 100 ^Aa^
	3	1893 ± 300 ^Aa^	1660 ± 83 ^Aab^	1735 ± 92 ^Aab^	1710 ± 100 ^Aab^	1560 ± 250 ^Aa^
	4	1393 ± 750 ^Aa^	1335 ± 158 ^Aab^	1035 ± 158 ^Aab^	618 ± 92 ^Ab^	1293 ± 633 ^Aa^
	5	243 ± 0 ^Ba^	527 ± 367 ^ABb^	518 ± 375 ^ABb^	410 ± 117 ^ABb^	1627 ± 50 ^Aa^
	6	385 ± 225 ^Aa^	459 ± 263 ^Aab^	560 ± 267 ^Ab^	302 ± 92 ^Ab^	602 ± 408 ^Aa^
Ascorbic acid content ([Ascorbic acid] mg/kg)	0	3280 ± 0 ^Aa^	3280 ± 0 ^Aa^	3280 ± 0 ^Aa^	3280 ± 0 ^Aa^	3280 ± 0 ^Aa^
	1	3200 ± 800 ^Aa^	3600 ± 400 ^Aa^	3200 ± 0 ^Aa^	2800 ± 400 ^Aab^	3600 ± 400 ^Aa^
	2	1600 ± 0 ^Aab^	1400 ± 200 ^Ab^	1400 ± 200 ^Abc^	1600 ± 0 ^Ac^	1400 ± 200 ^Ab^
	3	1600 ± 0 ^Aab^	1600 ± 0 ^Ab^	1600 ± 0 ^Abc^	1600 ± 0 ^Ac^	1600 ± 0 ^Ab^
	4	1600 ± 0 ^Cab^	1600 ± 0 ^Db^	1200 ± 0 ^Ec^	1600 ± 0 ^Bc^	1600 ± 0 ^Ab^
	5	1200 ± 0 ^Ab^	1600 ± 0 ^Ab^	1600 ± 0 ^Abc^	1400 ± 200 ^Ac^	1400 ± 200 ^Ab^
	6	1400 ± 200 ^Ab^	1600 ± 0 ^Ab^	1800 ± 200 ^Ab^	1800 ± 200 ^Abc^	1800 ± 0 ^Ab^
Betalains–Indicaxanthin (mg/100 g)	0	32.2 ± 0.0 ^Aa^	32.2 ± 0.0 ^Aa^	32.2 ± 0.0 ^Aa^	32.2 ± 0.0 ^Aa^	32.2 ± 0.0 ^Aa^
	1	30.8 ± 3.8 ^Aa^	31.4 ± 3.8 ^Aa^	33.0 ± 0.3 ^Aa^	30.8 ± 5.1 ^Aa^	34.3 ± 3.5 ^Aa^
	2	13.5 ± 3.2 ^Aa^	14.1 ± 3.2 ^Aab^	22.1 ± 2.2 ^Aa^	13.8 ± 4.8 ^Aa^	22.8 ± 1.6 ^Aa^
	3	31.8 ± 9.9 ^Aa^	29.5 ± 3.9 ^Aab^	36.3 ± 8.0 ^Aa^	26.0 ± 6.1 ^Aa^	26.6 ± 4.2 ^Aa^
	4	23.1 ± 6.4 ^Aa^	29.8 ± 4.2 ^Aa^	20.5 ± 5.1 ^Aa^	13.2 ± 2.9 ^Aa^	18.6 ± 7.7 ^Aa^
	5	21.5 ± 0.3 ^Aa^	16.4 ± 4.8 ^Aab^	24.7 ± 3.5 ^Aa^	24.1 ± 0.3 ^Aa^	25.7 ± 0.6 ^Aa^
	6	20.2 ± 6.7 ^Aa^	10.6 ± 1.0 ^Ab^	22.5 ± 0.6 ^Aa^	11.6 ± 1.9 ^Aa^	27.3 ± 16.4 ^Aa^
Betalains–Betanin (mg/100 g)	0	53.3 ± 0.0 ^Aa^	53.3 ± 0.0 ^Aa^	53.3 ± 0.0 ^Aa^	53.3 ± 0.0 ^Aa^	53.3 ± 0.0 ^Aa^
	1	48.6 ± 14.7 ^Aa^	53.6 ± 11.5 ^Aa^	51.8 ± 0.5 ^Aab^	52.3 ± 11.0 ^Aa^	60.5 ± 13.8 ^Aa^
	2	22.0 ± 5.5 ^Aa^	21.1 ± 6.4 ^Aa^	37.6 ± 1.8 ^Ab^	13.8 ± 7.3 ^Ab^	40.8 ± 7.8 ^Aa^
	3	58.2 ± 17.0 ^Aa^	50.0 ± 6.9 ^Aa^	65.1 ± 8.3 ^Aa^	43.1 ± 9.2 ^Aab^	43.1 ± 3.7 ^Aa^
	4	34.8 ± 11.9 ^Aa^	47.7 ± 9.2 ^Aa^	26.1 ± 6.0 ^Ab^	17.4 ± 3.7 ^Aab^	23.8 ± 8.2 ^Aa^
	5	31.6 ± 1.4 ^Aa^	24.8 ± 9.2 ^Aa^	37.6 ± 1.8 ^Ab^	38.0 ± 5.0 ^Aab^	36.2 ± 2.3 ^Aa^
	6	28.9 ± 14.2 ^Aa^	16.0 ± 0.5 ^Aa^	33.0 ± 7.3 ^Ab^	14.7 ± 1.8 ^Ab^	47.7 ± 33.0 ^Aa^

^(a–c)^: Within each parameter, values in the same column not sharing lower case superscript letters indicate statistically significant differences among weeks (*p* < 0.05); ^(A–E)^: Within each parameter, values in the same row not sharing upper case superscript letters indicate statistically significant differences among coatings (*p* < 0.05).

Ascorbic acid or vitamin C is present in fruits and plays an important role in contributing to the antioxidant capacity [76,80]. The red and orange fresh fruits presented a value of ascorbic acid of 3280 mg/kg and 1094 mg/kg, respectively. These values are similar to values reported for prickly pears by other authors, with the value obtained for the red fruit being slightly higher [100]. Regarding the red variety, a significant decrease in ascorbic acid content was observed after the first week across all treatments (*p* < 0.05), with control fruits showing the steepest reductions. Significant differences between coatings were also noted at week 4 when chitosan-coated samples presented a lower ascorbic acid content (1200 mg/kg), statistically different from other coatings. At week 6, starch (1800 mg/kg) and chitosan (1800 mg/kg) maintained significantly higher ascorbic acid content compared to the control (1400 mg/kg). In the orange variety, ascorbic acid levels remained relatively stable over time in all treatments, with no significant decline (*p* > 0.05). Alginate-, chitosan-, and starch-coated samples showed slightly higher retention of ascorbic acid by week 6 (1600 mg/kg), but the differences were not statistically significant when compared to the control (*p* > 0.05). At the end of 6 weeks, pectin-coated fruits demonstrated the lower value of ascorbic acid. Overall, ascorbic acid content was better preserved by coatings in the red variety, particularly with starch as a coating at the end of 6 weeks. The use of a pre-gelatinized corn starch/cellulose nanofiber/basil essential oil as a coating to preserve ascorbic acid content was also reported in mandarin oranges showing to be effective in the maintenance of this parameter after 12 days of storage [45]. Allegra et al. (2022) suggest that the use of an edible coating led to prickly pear protection from oxidation, leading to gas exchange reduction between the fruit and the environment [83]. The orange variety showed more stability over time even though the values were lower than the other variety, but no coating was particularly emphasized to preserve ascorbic acid content.

**Table 4:** Bioactive properties results for the coated and uncoated orange prickly pear pulp fruits during storage time.

Parameter	Week	Control	Alginate	Chitosan	Pectin	Starch
TPC (mg GAE/100 g)	0	588 ± 0 ^Aa^	588 ± 0 ^Aa^	588 ± 0 ^Aa^	588 ± 0 ^Aab^	588 ± 0 ^Aa^
	1	497 ± 57 ^Aa^	626 ± 51 ^Aa^	610 ± 24 ^Aa^	616 ± 89 ^Aab^	589 ± 19 ^Aa^
	2	654 ± 62 ^Aa^	535 ± 35 ^Aa^	548 ± 16 ^Aa^	621 ± 3 ^Aab^	583 ± 57 ^Aa^
	3	589 ± 41 ^Aa^	708 ± 14 ^Aa^	783 ± 197 ^Aa^	648 ± 51 ^Aab^	575 ± 81 ^Aa^
	4	516 ± 59 ^Aa^	467 ± 184 ^Aa^	667 ± 27 ^Aa^	505 ± 0 ^Ab^	443 ± 68 ^Aa^
	5	686 ± 176 ^Aa^	537 ± 59 ^Aa^	667 ± 38 ^Aa^	878 ± 108 ^Aa^	610 ± 46 ^Aa^
	6	564 ± 54 ^Aa^	486 ± 51 ^Aa^	662 ± 43 ^Aa^	567 ± 24 ^Aab^	635 ± 114 ^Aa^
Antioxidant activity (mg eq Trolox/100 mg)	0	4445 ± 54 ^Aa^	4445 ± 54 ^Aa^	4445 ± 54 ^Aa^	4445 ± 54 ^Aa^	4445 ± 54 ^Aa^
	1	685 ± 25 ^Abc^	910 ± 100 ^Abc^	777 ± 17 ^Abc^	727 ± 67 ^Ac^	852 ± 92 ^Ac^
	2	1543 ± 117 ^Ab^	1352 ± 142 ^ABb^	977 ± 50 ^Bb^	1277 ± 17 ^ABb^	1435 ± 75 ^ABb^
	3	760 ± 83 ^Abc^	602 ± 8 ^Acd^	552 ± 58 ^Acd^	560 ± 133 ^Acd^	377 ± 50 ^Ad^
	4	893 ± 417 ^Abc^	502 ± 75 ^Acd^	393 ± 100 ^Ad^	177 ± 33 ^Ade^	402 ± 58 ^Acd^
	5	110 ± 0 ^Bc^	185 ± 58 ^ABd^	685 ± 58 ^Abcd^	193 ± 67 ^ABde^	335 ± 158 ^ABd^
	6	180 ± 8 ^Abc^	138 ± 138 ^Ad^	368 ± 25 ^Ad^	352 ± 0 ^Ae^	460 ± 350 ^Ad^
Ascorbic acid content ([Ascorbic acid] mg/kg)	0	1094 ± 58 ^Aa^	1094 ± 58 ^Aa^	1094 ± 58 ^Aa^	1094 ± 58 ^Aa^	1094 ± 58 ^Aa^
	1	1200 ± 0 ^Aa^	1400 ± 200 ^Aa^	1400 ± 200 ^Aa^	1200 ± 0 ^Aa^	1400 ± 200 ^Aa^
	2	800 ± 0 ^Aa^	1200 ± 400 ^Aa^	1600 ± 0 ^Aa^	1600 ± 0 ^Aa^	1200 ± 400 ^Aa^
	3	1200 ± 400 ^Aa^	1400 ± 200 ^Aa^	1400 ± 200 ^Aa^	1600 ± 0 ^Aa^	1600 ± 0 ^Aa^
	4	1600 ± 0 ^Aa^	1600 ± 0 ^Ba^	1200 ± 0 ^Ea^	1200 ± 0 ^Da^	1200 ± 0 ^Ca^
	5	1200 ± 0 ^Aa^	1600 ± 0 ^Aa^	1800 ± 0 ^Aa^	1800 ± 200 ^Aa^	1600 ± 0 ^Aa^
	6	1600 ± 0 ^Aa^	1600 ± 0 ^Aa^	1600 ± 0 ^Aa^	1200 ± 400 ^Aa^	1600 ± 0 ^Aa^
Betalains–Indicaxanthin (mg/100 g)	0	20.9 ± 6.7 ^Aa^	20.9 ± 6.7 ^Aa^	20.9 ± 6.7 ^Aa^	20.9 ± 6.7 ^Aa^	20.9 ± 6.7 ^Aa^
	1	15.7 ± 3.5 ^Aa^	29.8 ± 11.9 ^Aa^	15.7 ± 2.2 ^Aa^	20.2 ± 4.2 ^Aa^	28.2 ± 3.2 ^Aa^
	2	24.4 ± 3.8 ^Aa^	21.2 ± 5.8 ^Aa^	30.8 ± 10.3 ^Aa^	21.2 ± 8.3 ^Aa^	34.3 ± 13.2 ^Aa^
	3	29.2 ± 1.6 ^Aa^	23.7 ± 6.4 ^Aa^	22.5 ± 1.3 ^A^	23.7 ± 1.3 ^A^	22.5 ± 6.4 ^Aa^
	4	24.7 ± 5.5 ^Aa^	23.1 ± 5.8 ^Aa^	31.8 ± 7.4 ^Aa^	24.4 ± 5.8 ^Aa^	23.4 ± 9.3 ^Aa^
	5	18.9 ± 0.3 ^Aa^	33.7 ± 17.0 ^Aa^	18.3 ± 5.5 ^Aa^	20.9 ± 1.6 ^Aa^	32.7 ± 3.9 ^Aa^
	6	20.2 ± 1.4 ^Aa^	12.5 ± 1.4 ^Aa^	16.4 ± 4.1 ^Aa^	11.2 ± 3.2 ^Aa^	19.3 ± 6.4 ^Aa^
Betalains–Betanin (mg/100 g)	0	12.83 ± 1.83 ^Aa^	12.83 ± 1.83 ^Aa^	12.83 ± 1.83 ^Aa^	12.83 ± 1.83 ^Aa^	12.83 ± 1.83 ^Aa^
	1	2.75 ± 0.92 ^Ab^	4.12 ± 1.38 ^Ab^	1.37 ± 1.37 ^Ab^	3.21 ± 1.38 ^Ab^	1.37 ± 0.46 ^Ab^
	2	0.46 ± 0.46 ^Ab^	1.38 ± 0.46 ^Ab^	1.38 ± 0.46 ^Ab^	1.38 ± 0.46 ^Ab^	1.83 ± 0.92 ^Ab^
	3	3.67 ± 0.00 ^Ab^	3.21 ± 0.46 ^Ab^	1.83 ± 1.83 ^Ab^	2.75 ± 0.00 ^Ab^	2.59 ± 0.46 ^Ab^
	4	5.04 ± 0.46 ^ABb^	4.12 ± 0.46 ^Bb^	6.42 ± 0.00 ^Aab^	4.58 ± 0.00 ^ABb^	5.04 ± 0.46 ^ABb^
	5	2.75 ± 0.92 ^Ab^	3.21 ± 1.38 ^Ab^	0.92 ± 0.92 ^Ab^	1.83 ± 0.00 ^Ab^	1.83 ± 0.92 ^Ab^
	6	4.13 ± 0.46 ^Ab^	3.67 ± 0.00 ^Ab^	4.58 ± 0.92 ^Ab^	3.21 ± 0.46 ^Ab^	3.67 ± 0.00 ^Ab^

^(a–e)^: Within each parameter, values in the same column not sharing lower case superscript letters indicate statistically significant differences among weeks (*p* < 0.05); ^(A–E)^: Within each parameter, values in the same row not sharing upper case superscript letters indicate statistically significant differences among coatings (*p* < 0.05).

Betalains are plant pigments that exhibit functional properties namely antioxidant capacity and in which prickly pears constitute a great source [99,104]. Betalains can be divided into two main groups, namely betacyanins (where betanin is included), of red-purple color, and betaxanthins (where indicaxanthin is included) of the yellow-orange color [104,105]. Indicaxanthin values for red and orange prickly pears were 32.2 mg/100 g and 20.9 mg/100 g, respectively, and are consistent with values reported in the literature [100]. In both red and orange prickly pear fruits, indicaxanthins did not exhibit significant differences (*p* > 0.05) between the different coatings and control throughout the study. In red fruits, it was possible to observe significant differences in alginate (*p* < 0.05) with a reduction at week 6 (10.6 mg/100 g) with a lower value when compared with the initial value (32.2 mg/100 g). In orange fruits at week 5, alginate and starch exhibited higher levels of indicaxanthin, but the differences were not statistically significant. At the end of the experiment, alginate and pectin-coated fruits showed lower values in indicaxanthin. In the red variety, starch can be suggested as the coating to help with the preservation of indicaxanthin when compared to the other coatings. The use of coatings was shown to be effective in the preservation of indicaxanthin in prickly pears coated with mucilage after 9 days of storage when compared with uncoated fruits [76]. The results suggest that in the orange variety, coatings did not provide any major benefit in the preservation of indicaxanthin content.

Betanin content in fresh fruits was 53.3 mg/100 g and 12.8 mg/100 g for red and orange varieties, respectively, being similar to values described by other authors [100]. Betanin content in red prickly pears was maintained relatively well, showing no statistically significant differences among coatings and control within the same time of experience. The same behavior was observed in orange fruits except for week 4, with significant differences (*p* < 0.05) between alginate- and chitosan-coated fruits. In red fruits, significant declines were observed by week 6, particularly in alginate- and pectin-coated samples (*p* < 0.05). Chitosan-coated samples had higher betanin retention (33 mg/100 g) and starch (47.7 mg/100 g) by week 6 compared to alginate (16.0 mg/100 g) and pectin (14.7 mg/100 g), making chitosan and starch potentially more effective coatings for betanin preservation. Despite starch-coated fruits showing higher values in betanin at the end of the sixth week of the experiment, it was not statistically significant when compared to the beginning (*p* > 0.05). Thus, chitosan appears to be the coating to be more effective in retaining betanin content. The use of mucilage from prickly pear as coating material of pricky pears was effective in the preservation of betanin content in prickly pears after 9 days of storage [76]. In orange fruits, betanin content dropped significantly after the start in all the coated and uncoated fruits (*p* < 0.05). At the end of the experiment, betanin content had stabilized at lower concentrations, with no significant differences between coatings (*p* > 0.05) showing that no coating was effective in maintaining betanin levels. Moreover, storage temperature, antioxidant compounds, and fruit maturity stage can produce effects on betalain content, leading to losses [106].

### 3.5. Microbiological Growth

Microbiological growth is an important parameter in terms of food safety since it is one of the major causes of food loss leading to economic losses [19,107]. In this experiment, mesophiles, yeast, and mold counts were evaluated through time in both peel and pulp for both varieties. For both varieties, there were some variations throughout the experiment, but it was observed that values of microbiological growth were lower in pulp when compared to peel. These general results not only emphasize the intrinsic protection function of the peel in fruits, but the use of coatings also played a significant role in avoiding higher contamination.

Mesophiles contamination in red prickly pear peel is presented in Table 5. Significant variations (*p* < 0.05) were noted between coatings during the entire experiment but not in the last week. At week 6, alginate- and pectin-coated fruits showed the lowest results for mesophile contamination but with no significant differences. The effects of alginate and pectin coatings were also demonstrated in blueberries [32].

Yeast and mold contamination in red prickly pear peels (*p* < 0.05) showed significant differences only in week 4. At the end of the experiment, both coated and control fruits showed values of yeast and mold contamination within the same range and starch-coated fruits were the ones that presented lower values. Starch coating proved to be potentially effective on delaying contamination of strawberries [108].

In red prickly pear pulp (Table 6), the contamination of mesophiles had some variations throughout the 6 weeks of the experiment. Significant differences were observed between coatings in weeks 1, 2, and 4. At the end of the experiment, even though no statistically significant differences (*p* > 0.05) were observed, control fruits showed the highest mesophilic contamination compared to coated fruits, and alginate- and chitosan-coated fruits showed the least contamination at the end, as was also demonstrated in kiwi fruits and sweet cherries [50,109]. Prickly pears showed no contamination in yeasts and molds at the beginning of the experiment, and those values increased over time. In the sixth week, all coatings showed a positive effect in protecting the fruits against contamination despite no significant differences found between them with chitosan showing the lower value. Chitosan demonstrated to be effective against yeasts and molds in fresh-cut kiwi fruit [109].

In the peel of orange prickly pears, some variations throughout the weeks had occurred (*p* < 0.05) (Table 7). In the sixth week, control fruits had a higher value of mesophiles when compared to coated fruits, even though no statistically significant differences were observed. Chitosan, pectin, and starch were the coatings that led to lower values of antimicrobial contamination in the peel of orange prickly pears. Pectin coating was also effective against mesophilic bacteria when applied to blueberries [32]. The intrinsic antimicrobial properties of chitosan demonstrated in this study were also reported in sweet cherries [50]. In terms of yeast and mold contamination, control fruits had an increase over time, showing statistically significant differences (*p* < 0.05), as did the coated fruits. Even though at the end of 6 weeks there were no significant differences observed, coatings apparently protected fruits against yeast and molds, especially chitosan. Chitosan coating was also effective against yeast and mold in sweet cherries [50].

In the pulp of orange prickly pears, the values in terms of mesophiles (Table 8) had some variations (*p* < 0.05) between coatings at weeks 2 and 3, but not for the rest of the weeks. By the end, starch-coated fruits showed lower values of mesophiles than the rest of the coated fruits and the control, which was also demonstrated in strawberries [108].

No significant statistical differences (*p* > 0.05) were observed in orange pulp regarding yeast and mold contamination at the end of the experiment (Table 8). Despite that, uncoated fruits showed the highest values of yeast and mold, indicating the possible protective effects of coatings. Chitosan- and starch-coated fruits had less contamination in the sixth week. Starch demonstrated the same tendency in strawberries [108], and chitosan in sweet cherries [50].

## 4. Conclusions

Coatings had a positive effect on the preservation of quality and shelf life of prickly pears kept under refrigeration for 6 weeks, with some differences among the red and orange varieties. The most successful coatings in the reduction of weight loss were starch in red prickly pears with a reduction of around 8% and chitosan in orange prickly pears with a reduction of around 10%, when compared with the control of both prickly pears that lost 23% and 18%, respectively. These coatings also contributed positively to the maintenance of the prickly pears’ color. In terms of texture, red prickly pears coated with pectin or alginate exhibited superior firmness, while orange prickly pears benefited most from chitosan or alginate coatings. Regarding bioactivity, starch was the best coating in preserving antioxidant activity in red prickly pears, while in orange prickly pears, the coatings did not produce a significant effect. Starch was also the most effective in maintaining ascorbic acid content, particularly in the red prickly pear. Starch also contributed to the preservation of indicaxanthin and betanin in red prickly pears that presented at the end of 6 weeks values of 27.3 mg/100 g and 47.7 mg/100 g, respectively, when compared to the initial values of 32.2 mg/100 g and 53.3 mg/100 g showing good retention of these bioactive compounds. In orange prickly pears, the starch-coated fruits also demonstrated superior efficacy in retaining both indicaxanthin and betanin content. Regarding microbiological quality, although all coated and control prickly pears showed similar levels of yeasts and molds, starch-coated fruits displayed lower contamination levels. In red prickly pears, alginate and pectin coatings were most effective at reducing antimicrobial contamination on the peel, and on the pulp alginate and chitosan showed lower levels of contamination (1.20 log CFU/g). Overall, starch and chitosan coatings showed the best results on the reservation of physicochemical, bioactive, and microbiological properties of prickly pears to meet consumers’ demand for fresh fruit. Even though this study focuses specifically on prickly pears, the fundamental principles behind the interaction between coating materials and fruit surfaces have broad relevance. Edible coatings create a barrier that minimizes moisture loss, gas exchange, and microbial growth, which are transversal challenges in fruit preservation. However, the effectiveness of these coatings varies depending on each fruit’s unique surface properties, respiration rates, and physicochemical characteristics. This research provides a valuable reference for extending the methodology application to other fruits, additional studies tailored to specific fruit types must be addressed to optimize formulations and validate their effectiveness.

The results of this study demonstrate the promising potential of coatings for preserving prickly pears, but several constraints must be addressed in future studies. A study of the impact of these coatings under different temperature conditions must be addressed. Additionally, the combination with other preservation methods, such as modified atmosphere, and the study of coating formulation in order to improve their features such as UV protection or antioxidant capacity, would be highly valuable for extending the shelf life of prickly pears even further. Future studies could also include the assessment of sensory qualities, such as taste and texture, to fully understand the effect of coatings on prickly pear quality from the consumer’s perspective. In addition, studies should investigate the scalability of these coatings for commercial use, taking into account the cost-effectiveness and practicality of applying these coatings in larger-scale operations. These areas of research would contribute to a more comprehensive understanding of how coatings can be used to enhance the shelf life and quality of prickly pears and similar fruits, while also addressing broader concerns related to sustainability and commercial viability.

## Figures and Tables

**Table 1:** Physicochemical results of the coated and uncoated red prickly pear pulp fruits during storage time.

Parameter	Week	Control	Alginate	Chitosan	Pectin	Starch
Moisture (%)	0	86.7 ± 2.2 ^Aa^	86.7 ± 2.2 ^Aa^	86.7 ± 2.2 ^Aa^	86.7 ± 2.2 ^Aa^	86.7 ± 2.2 ^Aa^
	1	82.2 ± 7.2 ^Aa^	82.0 ± 4.8 ^Aa^	85.0 ± 2.9 ^Aa^	84.1 ± 1.1 ^Aa^	84.7 ± 1.8 ^Aa^
	2	81.1 ± 2.4 ^Aa^	81.5 ± 2.0 ^Aa^	83.0 ± 0.9 ^Aa^	80.6 ± 0.4 ^Aa^	80.7 ± 1.6 ^Aa^
	3	79.4 ± 0.8 ^Aa^	79.8 ± 1.0 ^Aa^	81.9 ± 1.8 ^Aa^	79.8 ± 1.5 ^Aa^	80.3 ± 2.9 ^Aa^
	4	78.7 ± 1.0 ^Aa^	79.4 ± 0.0 ^Aa^	80.3 ± 1.1 ^Aa^	78.1 ± 0.5 ^Aa^	80.2 ± 2.8 ^Aa^
	5	77.8 ± 1.6 ^Aa^	78.8 ± 0.9 ^Aa^	80.3 ± 1.8 ^Aa^	76.4 ± 0.2 ^Aa^	78.8 ± 0.0 ^Aa^
	6	77.5 ± 0.2 ^Aa^	78.6 ± 2.5 ^Aa^	78.9 ± 1.7 ^Aa^	74.1 ± 9.8 ^Aa^	78.1 ± 1.6 ^Aa^
pH	0	6.15 ± 0.10 ^Aa^	6.15 ± 0.10 ^Aa^	6.15 ± 0.10 ^Aa^	6.15 ± 0.10 ^Aa^	6.15 ± 0.10 ^Aa^
	1	6.41 ± 0.04 ^Aa^	6.14 ± 0.16 ^Aa^	6.31 ± 0.04 ^Aa^	6.36 ± 0.05 ^Aa^	6.28 ± 0.01 ^Aa^
	2	6.31 ± 0.01 ^Aa^	6.26 ± 0.00 ^ABa^	6.18 ± 0.03 ^Ba^	6.29 ± 0.02 ^Aa^	6.23 ± 0.02 ^ABa^
	3	6.21 ± 0.04 ^Aa^	6.20 ± 0.00 ^Aa^	6.17 ± 0.17 ^Aa^	6.29 ± 0.04 ^Aa^	6.45 ± 0.08 ^Aa^
	4	6.11 ± 0.15 ^Aa^	6.19 ± 0.03 ^Aa^	6.21 ± 0.11 ^Aa^	6.15 ± 0.18 ^Aa^	6.17 ± 0.01 ^Aa^
	5	6.36 ± 0.05 ^Aa^	6.17 ± 0.29 ^Aa^	6.65 ± 0.02 ^Aa^	6.20 ± 0.12 ^Aa^	6.31 ± 0.11 ^Aa^
	6	5.96 ± 0.09 ^ABa^	5.53 ± 0.10 ^Ba^	6.14 ± 0.13 ^ABa^	6.08 ± 0.16 ^ABa^	6.39 ± 0.04 ^Aa^
TSS (°Brix)	0	13.2 ± 0.7 ^Aa^	13.2 ± 0.7 ^Aa^	13.2 ± 0.7 ^Aa^	13.2 ± 0.7 ^Aa^	13.2 ± 0.7 ^Aa^
	1	13.0 ± 2.0 ^Aa^	14.6 ± 0.4 ^Aa^	12.8 ± 1.8 ^Aa^	14.9 ± 0.8 ^Aa^	14.8 ± 0.1 ^Aa^
	2	14.7 ± 0.0 ^Aa^	16.5 ± 0.4 ^Aa^	12.7 ± 0.5 ^Aa^	14.0 ± 2.1 ^Aa^	11.9 ± 1.8 ^Aa^
	3	14.9 ± 0.8 ^Aa^	11.9 ± 0.6 ^Aa^	12.5 ± 2.0 ^Aa^	15.8 ± 0.4 ^Aa^	15.0 ± 0.1 ^Aa^
	4	11.7 ± 1.2 ^Aa^	12.3 ± 1.4 ^Aa^	12.4 ± 0.8 ^Aa^	10.7 ± 1.1 ^Aa^	12.1 ± 0.8 ^Aa^
	5	13.5 ± 1.3 ^Aa^	14.3 ± 1.5 ^Aa^	11.2 ± 0.9 ^Aa^	14.0 ± 2.6 ^Aa^	12.1 ± 3.4 ^Aa^
	6	11.6 ± 1.6 ^Aa^	13.1 ± 0.0 ^Aa^	12.7 ± 1.1 ^Aa^	13.0 ± 1.5 ^Aa^	13.2 ± 0.9 ^Aa^
Total titratable acidity (% citric acid (wt/wt))	0	0.024 ± 0.002 ^Ab^	0.024 ± 0.002 ^Ab^	0.024 ± 0.002 ^Ab^	0.024 ± 0.002 ^Ac^	0.024 ± 0.002 ^Aa^
	1	0.059 ± 0.004 ^Aa^	0.066 ± 0.010 ^Aab^	0.071 ± 0.008 ^Aa^	0.058 ± 0.002 ^Aab^	0.069 ± 0.010 ^Aa^
	2	0.052 ± 0.002 ^Aab^	0.061 ± 0.004 ^Aab^	0.054 ± 0.003 ^Aab^	0.053 ± 0.006 ^Aab^	0.063 ± 0.007 ^Aa^
	3	0.045 ± 0.005 ^Aab^	0.048 ± 0.006 ^Aab^	0.052 ± 0.010 ^Aab^	0.053 ± 0.002 ^Aab^	0.041 ± 0.005 ^Aa^
	4	0.048 ± 0.002 ^Aab^	0.051 ± 0.009 ^Aab^	0.056 ± 0.001 ^Aa^	0.052 ± 0.001 ^Ab^	0.054 ± 0.008 ^Aa^
	5	0.059 ± 0.008 ^Aa^	0.068 ± 0.020 ^Aab^	0.044 ± 0.006 ^Aab^	0.062 ± 0.008 ^Aab^	0.061 ± 0.026 ^Aa^
	6	0.061 ± 0.018 ^Aa^	0.083 ± 0.022 ^Aa^	0.052 ± 0.014 ^Aab^	0.069 ± 0.002 ^Aa^	0.057 ± 0.004 ^Aa^

^(a–c)^: Within each parameter, values in the same column not sharing lower case superscript letters indicate statistically significant differences among weeks (*p* < 0.05); ^(A,B)^: Within each parameter, values in the same row not sharing upper case superscript letters indicate statistically significant differences among coatings (*p* < 0.05).

**Table 2:** Physicochemical results of the coated and uncoated orange prickly pear pulp fruits during storage time.

Parameter	Week	Control	Alginate	Chitosan	Pectin	Starch
Moisture (%)	0	82.2 ± 0.1 ^Aa^	82.2 ± 0.1 ^Aa^	82.2 ± 0.1 ^Aa^	82.2 ± 0.1 ^Aa^	82.2 ± 0.1 ^Aa^
	1	81.2 ± 0.4 ^Aa^	82.2 ± 0.1 ^Aa^	80.9 ± 1.9 ^Aa^	82.2 ± 0.1 ^Aa^	82.2 ± 0.1 ^Aa^
	2	79.0 ± 0.7 ^Aa^	81.7 ± 1.1 ^Aa^	80.1 ± 1.5 ^Aa^	82.0 ± 1.8 ^Aa^	81.7 ± 2.8 ^Aa^
	3	77.4 ± 2.8 ^Aa^	79.8 ± 1.1 ^Aa^	79.9 ± 0.1 ^Aa^	80.8 ± 1.4 ^Aa^	80.5 ± 2.7 ^Aa^
	4	73.7 ± 2.6 ^Aa^	77.8 ± 2.8 ^Aa^	79.5 ± 0.1 ^Aa^	80.1 ± 1.4 ^Aa^	79.7 ± 1.4 ^Aa^
	5	69.0 ± 9.9 ^Aa^	77.6 ± 3.0 ^Aa^	78.9 ± 0.3 ^Aa^	78.4 ± 0.3 ^Aa^	79.0 ± 0.0 ^Aa^
	6	59.8 ± 22.4 ^Aa^	77.1 ± 0.1 ^Aa^	78.6 ± 0.2 ^Aa^	77.1 ± 0.7 ^Aa^	77.7 ± 0.7 ^Aa^
pH	0	6.48 ± 0.21 ^Aa^	6.48 ± 0.21 ^Aa^	6.48 ± 0.21 ^Aa^	6.48 ± 0.21 ^Aa^	6.48 ± 0.21 ^Aa^
	1	6.54 ± 0.04 ^Aa^	6.38 ± 0.01 ^Aa^	6.36 ± 0.07 ^Aa^	6.39 ± 0.06 ^Aa^	6.42 ± 0.00 ^Aa^
	2	6.33 ± 0.03 ^Aa^	6.36 ± 0.02 ^Aa^	6.35 ± 0.06 ^Aa^	6.30 ± 0.02 ^Aa^	6.37 ± 0.08 ^Aa^
	3	6.48 ± 0.05 ^Aa^	6.46 ± 0.04 ^Aa^	6.44 ± 0.05 ^Aa^	6.45 ± 0.01 ^Aa^	6.04 ± 0.64 ^Aa^
	4	6.34 ± 0.06 ^Aa^	6.47 ± 0.01 ^Aa^	6.25 ± 0.04 ^Aa^	6.51 ± 0.11 ^Aa^	6.40 ± 0.10 ^Aa^
	5	6.44 ± 0.02 ^Aa^	6.40 ± 0.02 ^Aa^	6.08 ± 0.18 ^Aa^	6.01 ± 0.21 ^Aa^	6.33 ± 0.21 ^Aa^
	6	6.43 ± 0.05 ^Aa^	6.36 ± 0.20 ^Aa^	5.98 ± 0.22 ^Aa^	6.36 ± 0.09 ^Aa^	6.45 ± 0.05 ^Aa^
TSS (°Brix)	0	13.5 ± 1.2 ^Aa^	13.5 ± 1.2 ^Aa^	13.5 ± 1.2 ^Aa^	13.5 ± 1.2 ^Aa^	13.5 ± 1.2 ^Aa^
	1	10.1 ± 1.3 ^Aa^	13.3 ± 0.1 ^Aa^	12.6 ± 0.6 ^Aa^	10.4 ± 0.0 ^Aa^	13.9 ± 0.7 ^Aa^
	2	10.2 ± 0.2 ^Aa^	13.6 ± 1.5 ^Aa^	10.5 ± 0.3 ^Aa^	12.0 ± 0.7 ^Aa^	11.8 ± 1.5 ^Aa^
	3	11.6 ± 1.6 ^Aa^	13.5 ± 0.3 ^Aa^	13.1 ± 0.6 ^Aa^	14.2 ± 0.1 ^Aa^	12.3 ± 0.9 ^Aa^
	4	16.8 ± 1.3 ^Aa^	14.2 ± 1.1 ^Aa^	13.4 ± 0.6 ^Aa^	11.4 ± 0.9 ^Aa^	13.2 ± 2.3 ^Aa^
	5	13.7 ± 0.8 ^Aa^	12.6 ± 0.1 ^Aa^	13.1 ± 0.7 ^Aa^	13.0 ± 0.9 ^Aa^	11.9 ± 1.4 ^Aa^
	6	12.7 ± 1.6 ^Aa^	12.0 ± 0.9 ^Aa^	11.3 ± 1.1 ^Aa^	12.9 ± 0.4 ^Aa^	11.6 ± 1.6 ^Aa^
Total titratable acidity (% citric acid (wt/wt))	0	0.025 ± 0.002 ^Ac^	0.025 ± 0.002 ^Ac^	0.025 ± 0.002 ^Ab^	0.025 ± 0.002 ^Ab^	0.025 ± 0.002 ^Aa^
	1	0.038 ± 0.002 ^Bbc^	0.050 ± 0.003 ^Aab^	0.043 ± 0.003 ^ABab^	0.047 ± 0.001 ^ABa^	0.052 ± 0.005 ^Aa^
	2	0.040 ± 0.003 ^Aab^	0.039 ± 0.003 ^Abc^	0.048 ± 0.006 ^Aab^	0.047 ± 0.004 ^Aa^	0.047 ± 0.000 ^Aa^
	3	0.042 ± 0.007 ^Aab^	0.046 ± 0.003 ^Aab^	0.053 ± 0.006 ^Aab^	0.047 ± 0.002 ^Aa^	0.056 ± 0.031 ^Aa^
	4	0.052 ± 0.001 ^Aab^	0.044 ± 0.007 ^Aab^	0.058 ± 0.012 ^Aab^	0.051 ± 0.008 ^Aa^	0.047 ± 0.006 ^Aa^
	5	0.045 ± 0.000 ^Aab^	0.055 ± 0.002 ^Aab^	0.058 ± 0.014 ^Aab^	0.064 ± 0.010 ^Aa^	0.052 ± 0.014 ^Aa^
	6	0.054 ± 0.004 ^Aa^	0.058 ± 0.006 ^Aa^	0.066 ± 0.014 ^Aa^	0.055 ± 0.006 ^Aa^	0.048 ± 0.004 ^Aa^

^(a–c)^: Within each parameter, values in the same column not sharing lower case superscript letters indicate statistically significant differences among weeks (*p* < 0.05); ^(A,B)^: Within each parameter, values in the same row not sharing upper case superscript letters indicate statistically significant differences among coatings (*p* < 0.05).

**Table 5:** Microbiological content results of the coated and uncoated red prickly pear peel fruits during storage time.

Parameter	Week	Control	Alginate	Chitosan	Pectin	Starch
Mesophiles (log CFU/g)	0	3.98 ± 0.26 ^Aa^	3.98 ± 0.26 ^Aa^	3.98 ± 0.26 ^Aa^	3.98 ± 0.26 ^Aa^	3.98 ± 0.26 ^Aa^
	1	3.45 ± 0.20 ^Aa^	3.76 ± 0.35 ^Aa^	3.76 ± 0.25 ^Aa^	3.76 ± 0.33 ^Aab^	4.60 ± 0.70 ^Aa^
	2	3.18 ± 0.38 ^Aa^	3.16 ± 0.73 ^Aa^	3.83 ± 0.65 ^Aa^	2.45 ± 0.20 ^Abc^	2.43 ± 0.48 ^Aa^
	3	3.69 ± 0.23 ^Aa^	4.37 ± 0.42 ^Aa^	3.72 ± 1.06 ^Aa^	2.55 ± 0.27 ^Abc^	2.99 ± 0.03 ^Aa^
	4	3.80 ± 0.75 ^Aa^	4.66 ± 0.65 ^Aa^	3.72 ± 0.72 ^Aa^	2.12 ± 0.09 ^Ac^	2.12 ± 0.39 ^Aa^
	5	2.90 ± 0.22 ^ABa^	3.66 ± 0.59 ^ABa^	2.40 ± 0.16 ^Ba^	3.21 ± 0.03 ^ABabc^	4.51 ± 0.41 ^Aa^
	6	3.29 ± 1.34 ^Aa^	2.66 ± 0.41 ^Aa^	3.68 ± 0.08 ^Aa^	2.93 ± 0.37 ^Aabc^	3.26 ± 0.53 ^Aa^
Yeasts and Molds (log CFU/g)	0	2.01 ± 0.02 ^Aa^	2.01 ± 0.02 ^Aa^	2.01 ± 0.02 ^Aa^	2.01 ± 0.02 ^Aa^	2.01 ± 0.02 ^Aa^
	1	3.04 ± 0.09 ^Aa^	3.04 ± 0.39 ^Aa^	3.56 ± 0.65 ^Aa^	3.13 ± 0.00 ^Aa^	3.75 ± 0.62 ^Aa^
	2	3.40 ± 0.12 ^Aa^	3.19 ± 0.06 ^Aa^	3.83 ± 0.84 ^Aa^	2.73 ± 0.30 ^Aa^	2.30 ± 0.35 ^Aa^
	3	3.10 ± 0.44 ^Aa^	3.79 ± 0.12 ^Aa^	4.09 ± 0.88 ^Aa^	3.41 ± 0.03 ^Aa^	3.44 ± 0.29 ^Aa^
	4	2.76 ± 0.33 ^ABa^	3.83 ± 0.23 ^Aa^	1.65 ± 0.00 ^Ba^	2.33 ± 0.68 ^ABa^	1.89 ± 0.24 ^ABa^
	5	3.17 ± 0.05 ^Aa^	3.45 ± 0.00 ^Aa^	2.28 ± 0.63 ^Aa^	3.43 ± 0.11 ^Aa^	3.85 ± 0.37 ^Aa^
	6	3.62 ± 1.49 ^Aa^	3.70 ± 0.97 ^Aa^	3.43 ± 0.37 ^Aa^	3.17 ± 0.21 ^Aa^	2.16 ± 0.50 ^Aa^

^(a–c)^: Within each parameter, values in the same column not sharing lower case superscript letters indicate statistically significant differences among weeks (*p* < 0.05); ^(A,B)^: Within each parameter, values in the same row not sharing upper case superscript letters indicate statistically significant differences among coatings (*p* < 0.05).

**Table 6:** Microbiological content results of the coated and uncoated red prickly pear pulp fruits during storage time.

Parameter	Week	Control	Alginate	Chitosan	Pectin	Starch
Mesophiles (log CFU/g)	0	2.00 ± 0.27 ^Aa^	2.00 ± 0.27 ^Aab^	2.00 ± 0.27 ^Aa^	2.00 ± 0.27 ^Aa^	2.00 ± 0.27 ^Aab^
	1	2.06 ± 0.07 ^Aa^	1.26 ± 0.00 ^ABb^	0.95 ± 0.21 ^Ba^	1.21 ± 0.28 ^ABa^	0.84 ± 0.11 ^Bb^
	2	3.43 ± 0.00 ^Aa^	2.14 ± 0.70 ^ABab^	1.74 ± 0.00 ^ABa^	0.96 ± 0.00 ^Ba^	1.59 ± 0.33 ^ABab^
	3	1.20 ± 0.24 ^Aa^	2.06 ± 1.10 ^Aab^	2.11 ± 0.25 ^Aa^	1.74 ± 0.18 ^Aa^	3.56 ± 0.94 ^Aa^
	4	1.46 ± 0.50 ^Ba^	4.48 ± 0.00 ^Aa^	1.95 ± 0.34 ^Ba^	1.65 ± 0.39 ^Ba^	0.95 ± 0.00 ^Bb^
	5	2.89 ± 0.53 ^Aa^	2.80 ± 0.36 ^Aab^	1.26 ± 0.00 ^Aa^	2.41 ± 0.75 ^Aa^	1.59 ± 0.33 ^Aab^
	6	2.69 ± 1.43 ^Aa^	1.20 ± 0.24 ^Ab^	1.20 ± 0.24 ^Aa^	1.26 ± 0.30 ^Aa^	1.74 ± 0.00 ^Aab^
Yeasts and Molds (log CFU/g)	0	<1 ^Aa^	<1 ^Ab^	<1 ^Ab^	<1 ^Ab^	<1 ^Aa^
	1	2.50 ± 0.00 ^Ba^	<1 ^Db^	<1 ^Db^	2.66 ± 0.00 ^Aa^	1.66 ± 0.00 ^Cc^
	2	3.53 ± 0.24 ^Aa^	1.96 ± 0.30 ^Bab^	2.26 ± 0.00 ^ABa^	2.16 ± 0.35 ^Ba^	2.20 ± 0.06 ^Bbc^
	3	1.66 ± 0.00 ^Aa^	2.83 ± 0.69 ^Aa^	1.96 ± 0.60 ^Aa^	2.16 ± 0.20 ^Aa^	3.27 ± 0.50 ^Aa^
	4	2.98 ± 0.25 ^Aa^	2.31 ± 0.05 ^ABab^	1.66 ± 0.00 ^Bab^	1.96 ± 0.00 ^ABa^	2.98 ± 0.45 ^Aab^
	5	2.78 ± 0.52 ^Aa^	2.67 ± 0.41 ^Aab^	1.84 ± 0.11 ^Aa^	2.61 ± 0.00 ^Aa^	1.66 ± 0.00 ^Abc^
	6	3.16 ± 1.50 ^Aa^	2.56 ± 0.00 ^Aab^	1.96 ± 0.00 ^Aa^	2.13 ± 0.00 ^Aa^	2.56 ± 0.00 ^Aab^

^(a–c)^: Within each parameter, values in the same column not sharing lower case superscript letters indicate statistically significant differences among weeks (*p* < 0.05); ^(A–D)^: Within each parameter, values in the same row not sharing upper case superscript letters indicate statistically significant differences among coatings (*p* < 0.05).

**Table 7:** Microbiological content results of the coated and uncoated orange prickly pear peel fruits during storage time.

Parameter	Week	Control	Alginate	Chitosan	Pectin	Starch
Mesophiles (log CFU/g)	0	3.69 ± 0.79 ^Aa^	3.69 ± 0.79 ^Aa^	3.69 ± 0.79 ^Aa^	3.69 ± 0.79 ^Aa^	3.69 ± 0.79 ^Aa^
	1	5.26 ± 0.00 ^Aa^	3.80 ± 0.55 ^ABa^	2.45 ± 0.21 ^Ba^	2.95 ± 0.00 ^Ba^	2.95 ± 0.00 ^Ba^
	2	3.04 ± 0.24 ^Aa^	2.92 ± 0.18 ^Aa^	2.72 ± 0.35 ^Aa^	2.65 ± 0.00 ^Aa^	2.45 ± 0.20 ^Aa^
	3	3.55 ± 0.39 ^Aa^	2.62 ± 0.25 ^ABa^	1.97 ± 0.17 ^Ba^	3.73 ± 0.00 ^Aa^	3.01 ± 0.05 ^ABa^
	4	4.01 ± 0.10 ^Aa^	3.10 ± 0.63 ^Aa^	2.31 ± 1.35 ^Aa^	3.77 ± 0.12 ^Aa^	2.83 ± 0.30 ^Aa^
	5	3.58 ± 0.21 ^ABa^	4.34 ± 0.07 ^Aa^	3.21 ± 0.00 ^ABa^	2.74 ± 0.49 ^Ba^	2.73 ± 0.00 ^Ba^
	6	5.28 ± 0.61 ^Aa^	3.54 ± 0.43 ^Aa^	3.14 ± 0.01 ^Aa^	3.00 ± 0.74 ^Aa^	3.11 ± 0.31 ^Aa^
Yeasts and Molds (log CFU/g)	0	1.61 ± 0.05 ^Ab^	1.61 ± 0.05 ^Ab^	1.61 ± 0.05 ^Acd^	1.61 ± 0.05 ^Ac^	1.61 ± 0.05 ^Ac^
	1	3.52 ± 0.09 ^ABab^	3.35 ± 0.00 ^Bab^	1.21 ± 0.00 ^Cd^	3.94 ± 0.00 ^Aa^	3.15 ± 0.20 ^Bab^
	2	3.30 ± 0.07 ^Aab^	2.75 ± 0.62 ^ABab^	1.56 ± 0.00 ^Bd^	3.53 ± 0.20 ^Aab^	3.39 ± 0.03 ^Aab^
	3	3.78 ± 0.07 ^Aa^	3.75 ± 0.06 ^Aa^	2.73 ± 0.00 ^Aa^	3.39 ± 0.39 ^Aab^	3.47 ± 0.10 ^Aab^
	4	3.78 ± 0.70 ^Aa^	3.65 ± 0.20 ^Aa^	2.12 ± 0.24 ^Abc^	2.52 ± 0.09 ^Abc^	2.65 ± 0.15 ^Ab^
	5	3.33 ± 0.17 ^Aab^	3.81 ± 0.23 ^Aa^	2.13 ± 0.00 ^Bb^	3.03 ± 0.23 ^ABab^	3.23 ± 0.06 ^Aab^
	6	5.07 ± 0.66 ^Aa^	4.39 ± 0.65 ^Aa^	2.47 ± 0.03 ^Aab^	3.34 ± 0.43 ^Aab^	3.54 ± 0.29 ^Aa^

^(a–d)^: Within each parameter, values in the same column not sharing lower case superscript letters indicate statistically significant differences among weeks (*p* < 0.05); ^(A–C)^: Within each parameter, values in the same row not sharing upper case superscript letters indicate statistically significant differences among coatings (*p* < 0.05).

**Table 8:** Microbiological content results of the coated and uncoated orange prickly pear pulp fruits during storage time.

Parameter	Week	Control	Alginate	Chitosan	Pectin	Starch
Mesophiles (log CFU/g)	0	2.67 ± 0.14 ^Aab^	2.67 ± 0.14 ^Aa^	2.67 ± 0.14 ^Aa^	2.67 ± 0.14 ^Aa^	2.67 ± 0.14 ^Aa^
	1	0.95 ± 0.00 ^Ab^	1.69 ± 0.26 ^Aa^	1.56 ± 0.30 ^Ab^	1.41 ± 0.15 ^Aab^	0.95 ± 0.00 ^Ab^
	2	2.21 ± 0.00 ^Cab^	2.24 ± 0.00 ^Ba^	1.80 ± 0.00 ^Db^	0.96 ± 0.00 ^Eb^	2.37 ± 0.00 ^Aab^
	3	2.55 ± 0.17 ^Aab^	1.97 ± 0.17 ^ABa^	1.55 ± 0.11 ^BCb^	0.96 ± 0.00 ^Cab^	1.94 ± 0.13 ^ABab^
	4	2.00 ± 0.00 ^Aab^	1.67 ± 0.24 ^Aa^	1.44 ± 0.00 ^Ab^	1.31 ± 0.35 ^Aab^	1.58 ± 0.33 ^Aab^
	5	3.01 ± 0.93 ^Aa^	1.35 ± 0.09 ^Aa^	1.44 ± 0.00 ^Ab^	0.96 ± 0.00 ^Aab^	1.46 ± 0.50 ^Aab^
	6	2.50 ± 0.00 ^Aab^	2.42 ± 1.16 ^Aa^	2.09 ± 0.13 ^Aab^	2.62 ± 0.71 ^Aa^	1.83 ± 0.27 ^Aab^
Yeasts and Molds (log CFU/g)	0	<1 ^Aa^	<1 ^Ac^	<1 ^Ab^	<1 ^Ab^	<1 ^Ab^
	1	2.13 ± 0.00 ^Aa^	<1 ^Ac^	<1 ^Ab^	1.66 ± 0.00 ^Aab^	2.16 ± 0.48 ^Aa^
	2	2.36 ± 0.00 ^ABa^	2.26 ± 0.00 ^Bb^	<1 ^Cb^	2.86 ± 0.00 ^Aa^	2.16 ± 0.20 ^Ba^
	3	2.46 ± 0.20 ^Aa^	2.01 ± 0.35 ^Ab^	2.44 ± 0.44 ^Aa^	2.56 ± 0.00 ^Aab^	2.13 ± 0.00 ^Aa^
	4	2.26 ± 0.00 ^Aa^	1.96 ± 0.30 ^Abc^	2.23 ± 0.27 ^Aa^	2.05 ± 0.09 ^Aab^	2.13 ± 0.00 ^Aa^
	5	2.70 ± 1.04 ^Aa^	2.26 ± 0.00 ^Ab^	1.66 ± 0.00 ^Aab^	1.66 ± 0.00 ^Aab^	<1 ^Ab^
	6	2.83 ± 0.70 ^Aa^	3.41 ± 0.00 ^Aa^	1.66 ± 0.00 ^Aab^	2.42 ± 0.76 ^Aab^	1.96 ± 0.00 ^Aab^

^(a–c)^: Within each parameter, values in the same column not sharing lower case superscript letters indicate statistically significant differences among weeks (*p* < 0.05); ^(A–E)^: Within each parameter, values in the same row not sharing upper case superscript letters indicate statistically significant differences among coatings (*p* < 0.05).

## Data Availability

The original contributions presented in this study are included in the article. Further inquiries can be directed to the corresponding author.
